# Supplementary material for: Microencapsulation of Lead-Halide Perovskites in an Oil-in-Fluorine Emulsion for Cell Imaging
Source: Nanomaterials (Basel). 2023 May 4;13(9):1540. doi: 10.3390/nano13091540 (PMC10180417; doi:10.3390/nano13091540)
Supplement: Supplementary file 1 [file nanomaterials-13-01540-s001.zip › nanomaterials-2341339-supplementary.pdf]

Supporting Information

Microencapsulation of Lead−halide Perovskites in an Oil−in−fluorine Emulsion for Cell Imaging

Jia-Xin Wang [1,2,§], Chang, Liu [3,§], Hao Huang [1], Rui He [1,], Shengyong Geng [1,*], and Xue-Feng Yu [1,2,3,*]*

1 Shenzhen Institute of Advanced Technology, Chinese Academy of Sciences, Shenzhen 518055, China

2 University of Chinese Academy of Sciences, Beijing 100049, China

3 Hubei Three Gorges Laboratory, Yichang 443007, China

* Correspondence: rui.he1@siat.ac.cn (R.H.); sy.geng@siat.ac.cn (S.G.); xf.yu@siat.ac.cn (X.-F.Y.)

† These authors contributed equally to this work.

Keywords: lead−halide perovskites; microencapsulation; oil−in−fluorine emulsion; cell imaging.

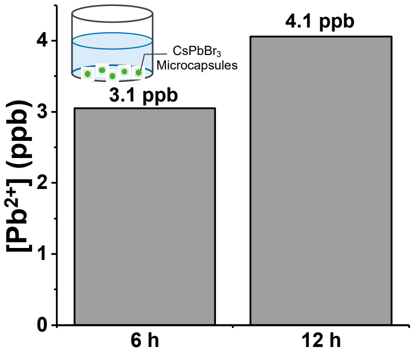

Figure S1. The leaked lead concentration after immersing the $CsPbBr_3$ microcapsules in deionized water for 6 h and 12 h. The inserted schematic illustration for lead leakage test.

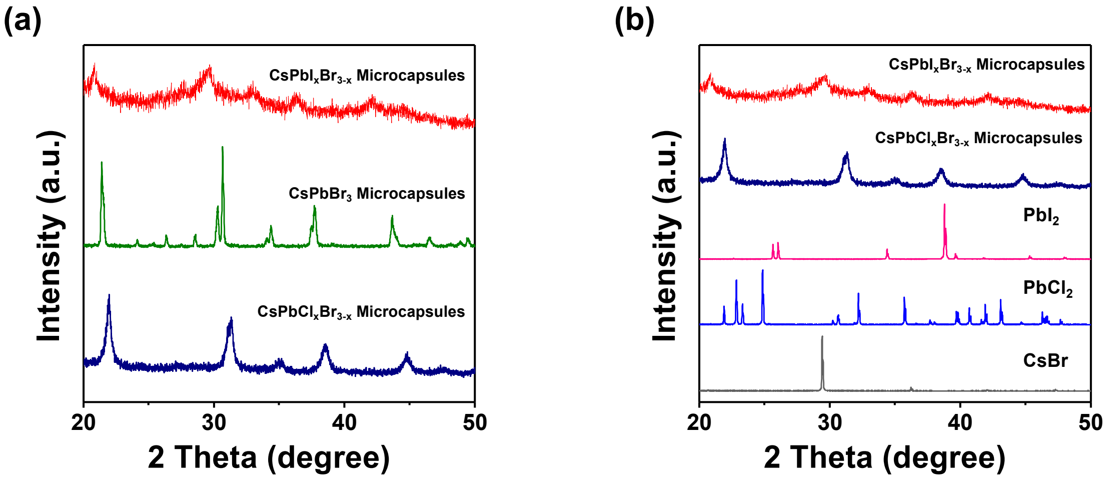

Figure S2. (**a**) XRD pattern for the $CsPbI_3Br_{3-x}$ microcapsules, $CsPbBr_3$ microcapsules and $CsPbCl_3Br_{3-x}$ microcapsules. As the halide ion was changed from Cl^- to Br^- and to I^-, the 2 theta angle shifts to a small angle direction. (**b**) XRD pattern for the $CsPbI_3Br_{3-x}$ microcapsules, $CsPbCl_3Br_{3-x}$ microcapsules, PbI_2, $PbCl_2$ and CsBr.

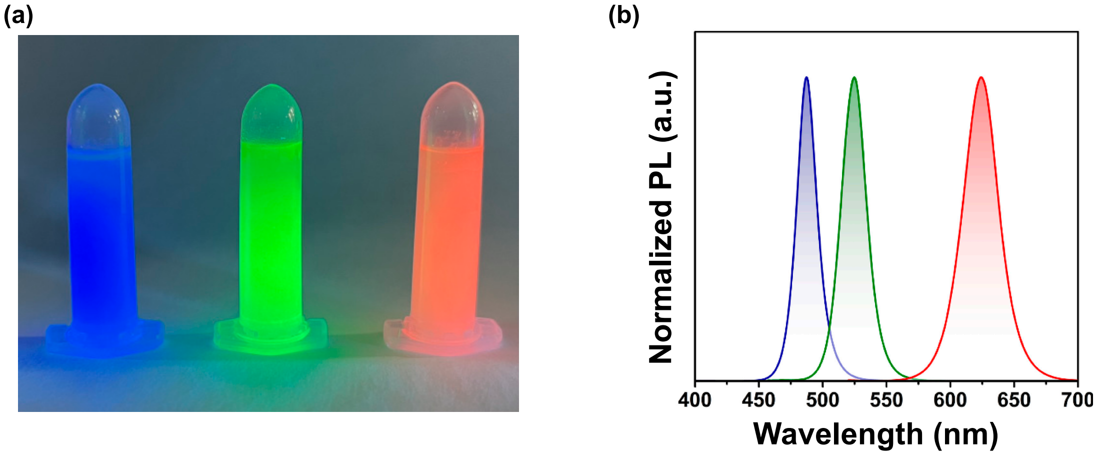

Figure S3. (**a**) Photograph and (**b**) PL emission spectra of LHP microcapsules with different emission

colors dispersed in PFO.

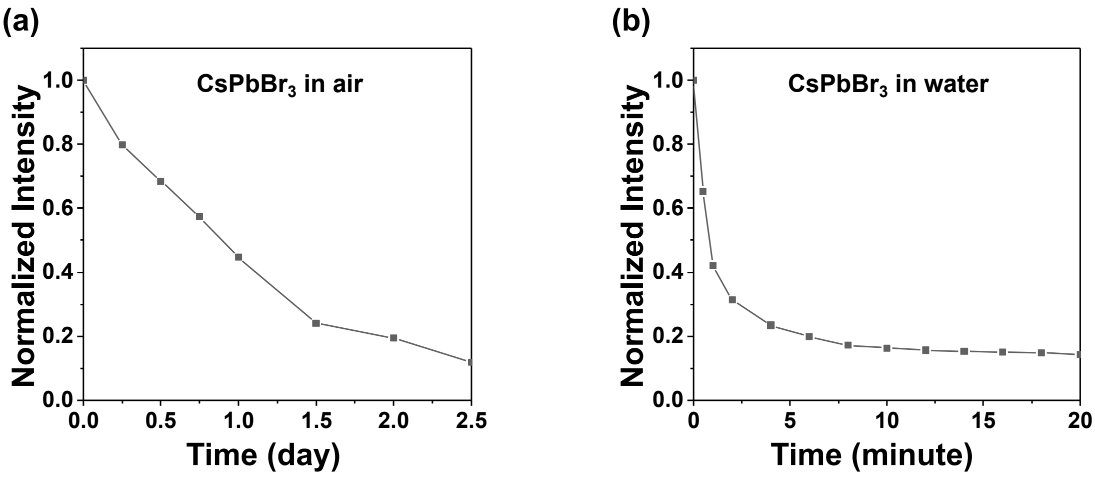

Figure S4. The relative fluorescence intensity of $CsPbBr_3$ as a function of time (**a**) in air and (**b**) in water.

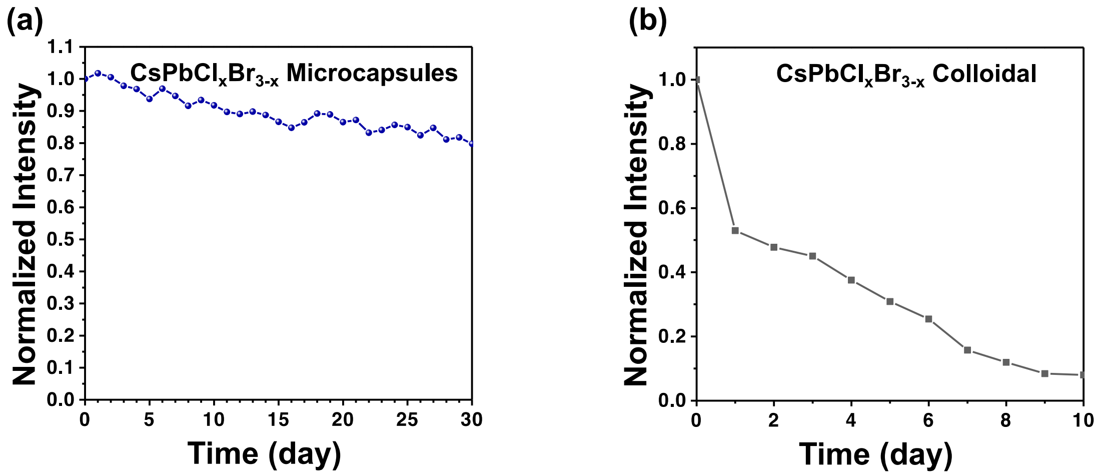

Figure S5 The relative fluorescence intensity of (**a**) $CsPbCl_xBr_{3-x}$ microcapsules and (**b**) colloidal $CsPbCl_xBr_{3-x}$ as a function of air exposure.

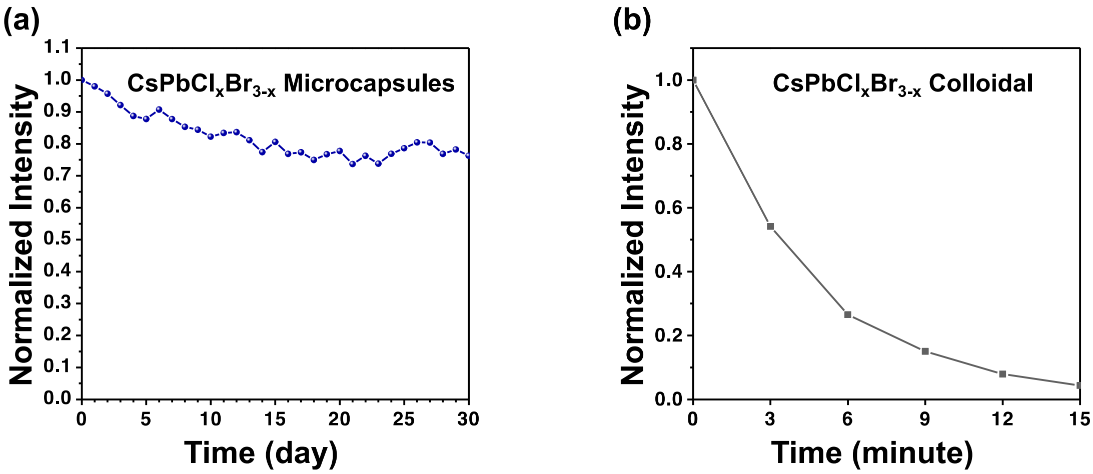

Figure S6. The relative fluorescence intensity of (**a**) $CsPbCl_xBr_{3-x}$ microcapsules and (**b**) colloidal $CsPbCl_xBr_{3-x}$ as a function of water exposure.

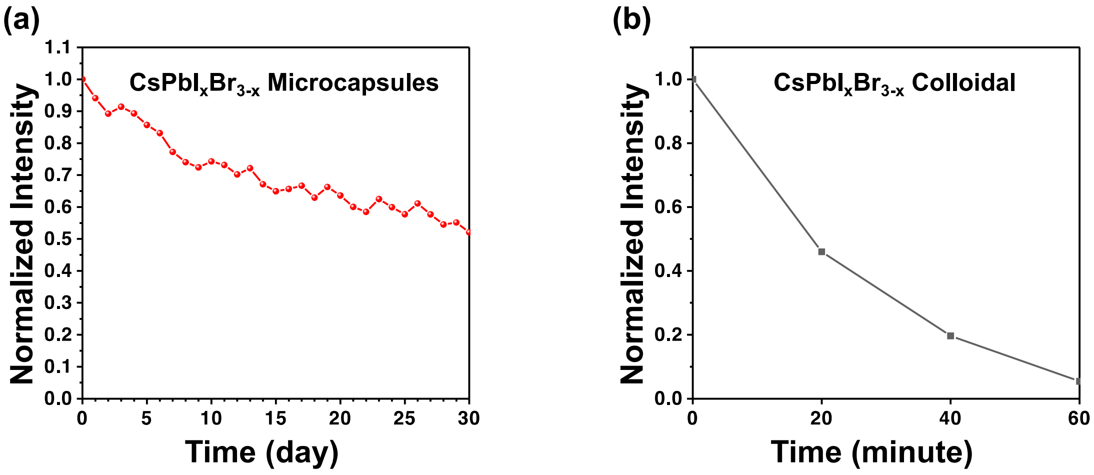

Figure S7. The relative fluorescence intensity of (**a**) $CsPbI_xBr_{3-x}$ microcapsules and (**b**) colloidal $CsPbI_xBr_{3-x}$ as a function of air exposure.

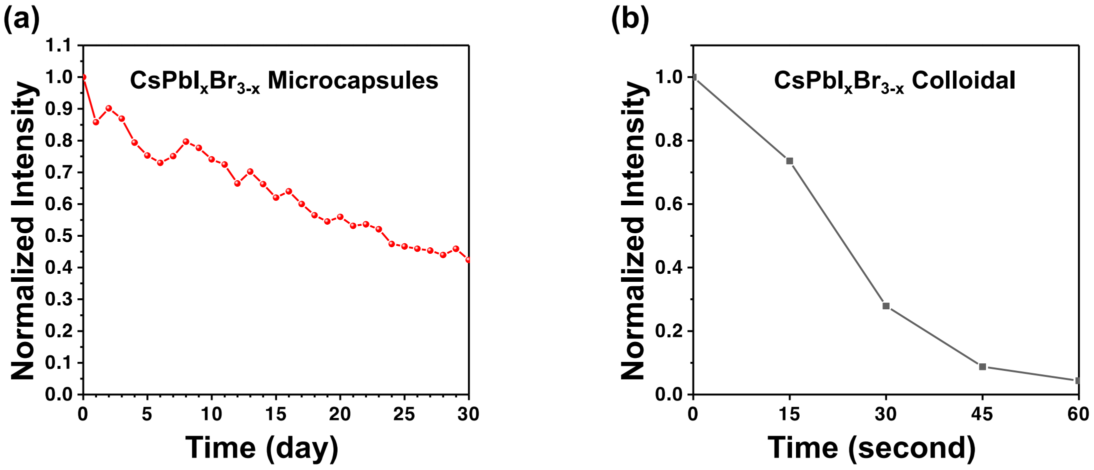

Figure S8. The relative fluorescence intensity of (**a**) $CsPbI_xBr_{3-x}$ microcapsules and (**b**) colloidal $CsPbI_xBr_{3-x}$ as a function of time in the water.

Table S1. Tabulated Fitted Lifetime Components for the $CsPbBr_3$ microcapsules and pure $CsPbBr_3$ perovskites.

Materials	τ_1	τ_2	$\chi 2$	τ_{avg}
**$CsPbBr_3$ microcapsules**	**3.83 ns (61.2%)**	**19.71 ns (38.7%)**	**0.994**	**15.98 ns**
**pure $CsPbBr_3$ perovskites**	**2.48 ns (75.8%)**	**13.66 ns (24.2%)**	**0.995**	**9.61 ns**
